# An In Vitro Model of Antibody-Enhanced Killing of the Intracellular Parasite *Leishmania amazonensis*

**DOI:** 10.1371/journal.pone.0106426

**Published:** 2014-09-05

**Authors:** Katherine N. Gibson-Corley, Marie M. Bockenstedt, Huijuan Li, Paola M. Boggiatto, Yashdeep Phanse, Christine A. Petersen, Bryan H. Bellaire, Douglas E. Jones

**Affiliations:** 1 Department of Veterinary Pathology, College of Veterinary Medicine, Iowa State University, Ames, Iowa, United States of America; 2 Department of Veterinary Microbiology and Preventive Medicine, College of Veterinary Medicine, Iowa State University, Ames, Iowa, United States of America; Federal Institute for Vaccines and Biomedicines, Germany

## Abstract

Footpad infection of C3HeB/FeJ mice with *Leishmania amazonensis* leads to chronic lesions accompanied by large parasite loads. Co-infecting these animals with *L. major* leads to induction of an effective Th1 immune response that can resolve these lesions. This cross-protection can be recapitulated in vitro by using immune cells from *L. major*-infected animals to effectively activate *L. amazonensis*-infected macrophages to kill the parasite. We have shown previously that the B cell population and their IgG2a antibodies are required for effective cross-protection. Here we demonstrate that, in contrast to *L. major*, killing *L. amazonensis* parasites is dependent upon FcRγ common-chain and NADPH oxidase-generated superoxide from infected macrophages. Superoxide production coincided with killing of *L. amazonensis* at five days post-activation, suggesting that opsonization of the parasites was not a likely mechanism of the antibody response. Therefore we tested the hypothesis that non-specific immune complexes could provide a mechanism of FcRγ common-chain/NADPH oxidase dependent parasite killing. Macrophage activation in response to soluble IgG2a immune complexes, IFN-γ and parasite antigen was effective in significantly reducing the percentage of macrophages infected with *L. amazonensis*. These results define a host protection mechanism effective during *Leishmania* infection and demonstrate for the first time a novel means by which IgG antibodies can enhance killing of an intracellular pathogen.

## Introduction

Infection of mice with the protozoal parasite *Leishmania* sp. is a useful model to understand the host-pathogen relationship. This system has defined host mechanisms required for an effective leishmanicidal response as well as how parasites limit host responses. Interestingly, some mouse strains are resistant to one *Leishmania* parasite species or strain, yet susceptible to another [1], [2], [3], [4]. In several different experimental models, infection with a *Leishmania* parasite to which the host mounts a healing immune response, such as *L. major*, induces a cross-protective immune response against a parasite to which the host is normally susceptible, such as *L. amazonensis* [5], [6], [7], [8]. Here we use an in vitro model of *L. major*-induced cross-protection against *L. amazonensis* infection. In this system, cross-protection was dependent upon antibody scavenger receptor signaling and NADPH oxidase activation. Furthermore, we find that non-specific soluble immune complexes (ICs) promoted a NADPH oxidase-dependent leishmanicidal response post-infection. These results define a host protection mechanism effective during *Leishmania* infection and demonstrate for the first time a novel means by which IgG antibodies can enhance killing of an intracellular pathogen.

Murine *L. amazonensis* infection generates a fundamentally different host response when compared to *L. major* infection [3], [9], [10], [11]. The response is characterized by a poor initial inflammatory response and ineffective T cell-mediated immunity [2], [12]. These results correspond to specific effects on antigen presenting cell (APC) function during infection that could, in part, account for the poor adaptive immune response associated with this parasite [13], [14]. However, several studies have demonstrated that simply enhancing a Th1 response either through immune modulation or using animals deficient in IL-10 has, at most, only modest effects on the long-term disease outcomes [2], [15], [16], [17]. Differences between *L. amazonensis* and *L. major* are also readily apparent using in vitro assays. *L. amazonensis* was shown to be more resistant to nitric oxide when compared to *L. major* [18], [19]. Additionally, IFN-γ, in contrast to being an effective molecule for macrophage activation, was shown to promote *L. amazonensis* replication within macrophages unless paired with other activating molecules [17]. These facts suggest that additional microbicidal pathways beyond nitric oxide production are required for immune control of *L. amazonensis* and therefore this protozoan parasite serves as a model system for identifying mechanisms of parasite control beyond the IFN-γ/nitric oxide response.

Successful macrophage activation secondary to parasite infection consists of production of microbicidal molecules. In experimental *Leishmania* infection of mice the central effector molecule is nitric oxide [20]. A summation of our understanding of productive immunity to this genus of parasite is that cell-mediated immunity is required, culminating in the production of IFN-γ from antigen specific CD4^+^ T cells and nitric oxide by infected macrophages [21]. However, additional microbicidal mechanisms such as nicotinamide adenine dinucleotide phosphate reduced form (NADPH) oxidase-dependent superoxide production can be important effector molecules against *Leishmania* spp. or strains that are resistant to the antimicrobial effects of nitric oxide [18], [19]. NADPH oxidase is a membrane bound enzyme complex which, when activated, generates the reactive free radical superoxide. This complex can be activated by an FcγR-mediated mechanism, which is dependent upon PI3 kinase (PI3K) signaling [22]. Activation of NADPH oxidase is highly regulated and dependent upon the appropriate assembly of the enzyme's cytosolic components p47^phox^, p67^phox^, p40^phox^ with the membrane-restricted components gp91^phox^ and p22^phox^ [23]. Inhibition of NADPH oxidase function appears to be a virulence strategy of several *Leishmania* sp. including *L. amazonensis*, *L. pifanoi*, and *L. donovani* [24], [25], [26]. FcγR-mediated activation at the macrophage cell membrane can be a potent mechanism to drive NADPH oxidase function either via phagocytosis or direct activation using FcγRI agonists [23], [27], [28], [29]. FcγRI-mediated superoxide production is dependent upon targeting of the p40^phox^ cytosolic subunit of NADPH oxidase to phosphatidylinositol-3-phosphate positive membranes, which are generated by a phosphatidylinositol 3-kinase (PI3K) [23], [30].

*L. amazonensis/L. major* co-infection in C3H mice can be used as a model system to determine what additional mechanisms of immunity are generated during co-infection that promote effective immunity against *L. amazonensis*. We previously demonstrated that CD4^+^ T cells and B cells, harvested from *L. major*-infected animals and restimulated with antigen ex vivo, provided the required immune factors to significantly enhance the microbicidal response of *L. amazonensis*-infected macrophages [31]. Antibodies, in particular the IgG2a isotype, and superoxide production were required to kill *L. amazonensis* [31], [32]. Fcγ receptors (FcγR), a component of several immunoglobulin scavenger receptors, are membrane bound receptors present on many cell types, including macrophages, and bind the Fc portion of antibodies which signal downstream events such as phagocytosis and/or cytotoxic activity. We wanted to test the hypothesis that superoxide production was linked to FcRγ-common chain signaling and NADPH oxidase. Upon confirmation that the microbicidal response was dependent upon FcRγ function we tested the hypothesis that soluble IgG2a immune complexes can activate *L. amazonensis*-infected macrophages to kill the parasite.

## Materials and Methods

### Mice

Female C57BL/6 (B6) mice, IFN-γ receptor (IFN-γR) knockout mice, inducible nitric oxide synthase (iNOS) deficient mice and gp91^phox^ deficient mice (all on B6 background) (6–8 weeks of age) were obtained from Jackson Laboratories (Bar Harbor, Maine). Fc receptor γ-common chain (FcRγ) knockout mice on a B6 background were kindly donated by Dr. Mary Anne McDowell, University of Notre Dame. Female C3HeB/FeJ (C3H) (6–8 weeks of age) were obtained from an in-house breeding colony. Mice were maintained in a specific pathogen-free facility. To obtain lymph node (LN) cells from infected animals, C3H mice were challenged with 5×10^6^ stationary-phase promastigotes in 50 µl of PBS in the left hind footpad. For the propagation of lesion-derived amastigotes female C3H severe combined immunodeficient (SCID) mice were infected with 2×10^7^ stationary-phase promastigotes in 50 µl of PBS in the left hind footpad. Ethics statement: All procedures involving animals were approved by the institutional animal care and use committee at Iowa State University in accordance with the Guide for the Care and Use of Laboratory Animals of the National Institutes of Health under protocol number 9-2-5266. Animals were euthanized by CO_2_ overdose in an enclosed chamber. All animals were monitored weekly to ensure lesions did not become ulcerated, necrotic or secondarily infected and cause distress.

### Parasites and antigens

*L. amazonensis* (MHOM/BR/0016/LTB) and *L. major* (MHOM/IL/80/Friedlin) promastigotes were grown in complete Grace's Insect medium (Atlanta Biologicals, Lawrenceville, GA) to stationary phase, harvested, washed in endotoxin-free PBS (Cellgro, Herdon, VA) and prepared to a concentration of 1×10^8^ parasites per milliliter. *L. major* and *L. amazonensis* amastigotes were collected from SCID mouse infected footpads. Freeze-thawed *Leishmania* antigen (Ft-Ag) was obtained from stationary-phase promastigotes as described [16].

### Bone marrow macrophages and cell culture

Bone marrow cells were harvested from femurs and tibias of C3H, B6 or genetically modified mice (1–3 mice per experiment) and plated in 150×15 mm Petri dishes with 30 ml of macrophage medium (30% L-cell conditioned medium, 20% fetal bovine serum (FBS), 50% Dulbecco's modification of eagle's medium (DMEM), 2 mM L-glutamine, 100 U penicillin per ml, 100 µg of streptomycin per ml and 1 mM sodium pyruvate) at 37°C and 5% CO_2_, after 2 days an additional 20 ml of macrophage medium was added. At day 7, the adherent cell population was collected and, after washing with PBS, trypan blue exclusion was used to count live cells, which were resuspended in complete tissue culture medium (CTCM; DMEM, 2 mM L-glutamine, 100 U penicillin, 100 µg streptomycin/ml, 25 mM HEPES, 0.05 um 2-mercaptoethanol and 10% FBS).

### Macrophage infection and treatments

Bone marrow-derived macrophages (BMM) were infected with either amastigotes or promastigotes as indicated in the figure legends at 3∶1 parasite to cell ratio. In indicated experiments *L. amazonensis* promastigotes were labeled with carboxyfluorescein diacetate succinimidyl ester (CFSE). The infected cells were incubated on coverslips at 34°C with 5% CO_2_ in 24 well plates. After 24 hours, macrophages were washed with warm DMEM to remove extracellular parasites and brought to a final volume of 0.5 ml with CTCM.

### Lymph node cell culture

Total LN cells were obtained from the left popliteal LN draining the site of infection from C3H mice infected for 4 weeks with *L. major*. LNs from 10–15 mice were pooled into 2 ml of CTCM and a single cell suspension was created using a 2 ml tissue grinder. Cells were washed with 10 ml of CTCM at 250 g, 4°C for 10 minutes. Following washing, these cells were resuspended in 5 ml CTCM, passed through 40 mm nylon cell strainers (BD Falcon, Bedford, MA) and counted via trypan blue exclusion. The total LN cells were added to the top compartment of 0.4 µm diameter transwell along with freeze-thawed *L. major* promastigote antigen as previously described [31]. Where indicated, co-cultures were incubated with 100 nM wortmannin (Sigma, St. Louis, MO) at 37°C for the time indicated.

### Activation of infected macrophages using soluble immune complexes

Mouse IgG2a κ isotype (functional grade purified, eBioscience) was labeled with Alexa Fluor (AF) 647 (Invitrogen). Soluble immune complexes (ICs) were formed by combining mouse IgG2a and goat anti-mouse IgG F(ab′)_2_ (AffiniPure F(ab′)_2_ Fragment, Jackson ImmunoResearch) at a 2∶1 molar ratio and incubated for 2 hours at 37°C with 5% CO_2_. ICs were centrifuged at 15,000 g for 10 minutes and the supernatant was collected for use [33]. Twenty-four hours following infection with *L. amazonensis* promastigotes, macrophages were washed with warm DMEM and the media was replaced with 0.5 ml CTCM. Cells were then incubated with these pre-formed soluble ICs, IFN-γ(Peprotech), FT-Ag, apocynin (Sigma), as indicated. Cells were then incubated for 4 days at 34°C with 5% CO_2_. On day 4, cells were harvested using Trypsin EDTA and fixed with 1% paraformaldehyde.

### Determination of macrophage infection rate

Following incubation for 5 days, coverslips were harvested, fixed and stained using nonspecific HEMA 3 stain set (Fisher Diagnostics, Middletown, VA). Coverslips were mounted onto glass slides and counted via light microscopy at 100x oil magnification. Three areas of 100 cells each were examined and the number of infected macrophages/100 cells was recorded. Treatments were blinded to the individual counting the coverslips.

### Determination of superoxide

Production of superoxide was assessed using Nitro Blue Tetrazolimide (NBT) (Sigma, St. Louis, MO) tablets. NBT tablets were dissolved in 1 ml sterilized deionized water and 30 ml of NBT was added to designated wells and incubated for 60–90 minutes at 37°C, 5% CO_2_. Coverslips were then harvested, fixed and stained with eosin and examined under the microscope for formazan precipitates.

### Immunofluorescence

Following incubation for the designated number of days, coverslips with adherent BMM were harvested and fixed with 4% paraformaldehyde in PBS for 20 minutes at room temperature and washed three times with PBS. BMM were permeabilized with 0.01% Triton X in PBS for 10 minutes at room temperature. Cells were incubated overnight at 4°C with goat anti-mouse gp91-phox and rabbit anti-mouse p67-phox (Santa Cruz Biotechnology, Santa Cruz, CA) at a 1∶50 and 1∶100 dilution in PBS, respectively. After incubation, coverslips were washed three times with PBS and incubated for 1 hour at room temperature with anti-goat Cy3-conjugated antibody or anti-rabbit Cy2-conjugated antibody, respectively (Jackson ImmunoResearch Laboratories, West Grove, PA) at a 1∶200 dilution in PBS. BMM were counterstained with 4′6-Diamidino-2-phenindole (DAPI) according to manufacturer's instructions (Sigma, St. Louis, MO). Coverslips were mounted onto slides using MOWIOL (Calbiochemical, La Jolla, CA) and viewed by sequential scanning confocal microscopy using an Olympus IX81 inverted scope (Olympus America Inc., Center Valley, PA). Quantitative co-localization analysis was performed with Olympus Fluoview version 2.1b software. A Pearson's correlation coefficient was calculated for channel 1 and channel 2 pixel intensity for each of >100 cells per treatment group and then averaged.

### ImageStream

To determine the percent of BMM infected with *L. amazonensis*, BMM were harvested and fixed at day 4 and analyzed by multispectral imaging flow cytometry (MIFC). This was based on previously published protocol with some modifications [34]. Briefly, day 4 treated macrophages were fixed with 1% paraformaldehyde in PBS. Sample acquisition was performed using Imagestream^x^ (Amnis corporation, Seattle) with a 488 nm laser. Samples were analyzed using IDEAS v.5.0 software (Amnis corporation). Signals from bright field, CFSE, and AF 647 were collected in channels 1, 2, and 5, respectively. The population of focused and single cells was selected for analysis. Parasite positive cells were gated by identifying CFSE positive cells on intensity and a maximum pixel fluorescence above background. The accuracy of this gating strategy was verified by examining the CFSE images from inside and outside the gated region. This gate was used to determine the percentage of parasite positive cells per single/focused cell population. The average number of parasites per cell was determined by using the IDEAS guided analysis for a spot count consistent with parasites visible via bright field. The number of spots per cell was then determined. To determine co-localization the percentage of AF 647 positive, CFSE positive spots were determined.

### Statistical Procedure

Statistical analysis was performed with Statview (SAS, Cary, NC) using ANOVA with Scheffe's multiple comparison or Graph Pad's Tukey-Kramer multiple comparisons or Dunnett's comparison post-hoc tests as indicated in the figure legends. Differences were considered significant when p<0.05.

## Results

### Immune cell-mediated killing of *L. amazonensis* requires NADPH oxidase and FcRγ-common chain

**Figure 1:**
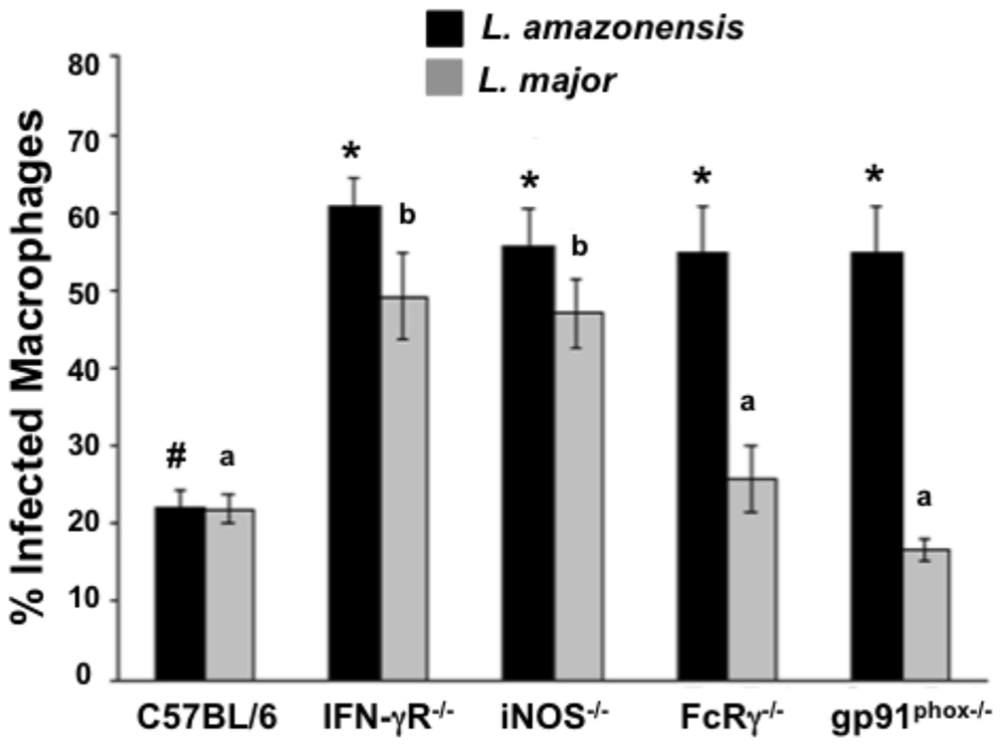
*L. amazonensis-*infected macrophages require IFN-γR, iNOS, FcRγ-common chain, and gp91^phox^ for parasite killing. BMM were infected with *L. amazonensis* or *L. major* amastigotes for 24 h and co-cultured with total lymph node cells from *L. major*-infected mice. After 5 days coverslips were stained with HEMA 3 and examined by microscopy to determine the percentage of BMM containing parasites. C57BL/6 =  wild-type, IFNγR^-/-^  =  Interferon-gamma receptor deficient BMM, iNOS^-/-^  =  inducible nitric oxide synthase deficient BMM, FcRγ^-/-^  =  Fcγ receptor-common chain deficient BMM, and gp91^phox^^-/-^ deficient BMM. For *L. amazonensis* infection different symbols represent a statistically significant difference (p<0.001) and for *L. major* infection different letters indicate a statistically significant difference (p<0.05). Results are the mean +/− SEM of three separate experiments. Statistical analysis was performed with Statview (SAS, Cary, NC) using ANOVA with Scheffe's multiple comparison.

Both antigen-specific CD4^+^ T cells and iNOS are required and, together, sufficient for immune control of *L. major* infection [21]. However, our previous studies using cell isolation experiments determined that both CD4^+^ T cells and CD19^+^ cells (B cells) from *L. major*-infected mice were needed to kill *L. amazonensis* within infected macrophages and IgG2a antibodies were required for this parasite killing [31], [32]. We hypothesized that these antibodies bind to stimulatory FcγR, which in turn activate NADPH oxidase production of superoxide. To test this hypothesis we used BMM (bone marrow-derived macrophages) from wild-type C57BL/6 mice along with BMM from IFN-γR, iNOS, FcRγ-common chain, and gp91^phox^ deficient mice. As expected, when co-cultured with total lymph node cells from *L. major*-infected mice, BMM from mice genetically deficient in IFN-γR and iNOS did not kill *L. major*, whereas this parasite was killed in BMM from FcRγ-common chain or gp91^phox^ deficient mice (Fig. 1, gray bars), as neither antibodies nor NADPH oxidase has been implicated in the immune control of *L. major* [21]. In contrast, under identical activation conditions, none of the genetically deficient BMM could kill *L. amazonensis*, including those from FcRγ-common chain or gp91^phox^ deficient mice (Fig. 1, black bars).

### NADPH oxidase-dependent superoxide is produced at the time of parasite killing

**Figure 2:**
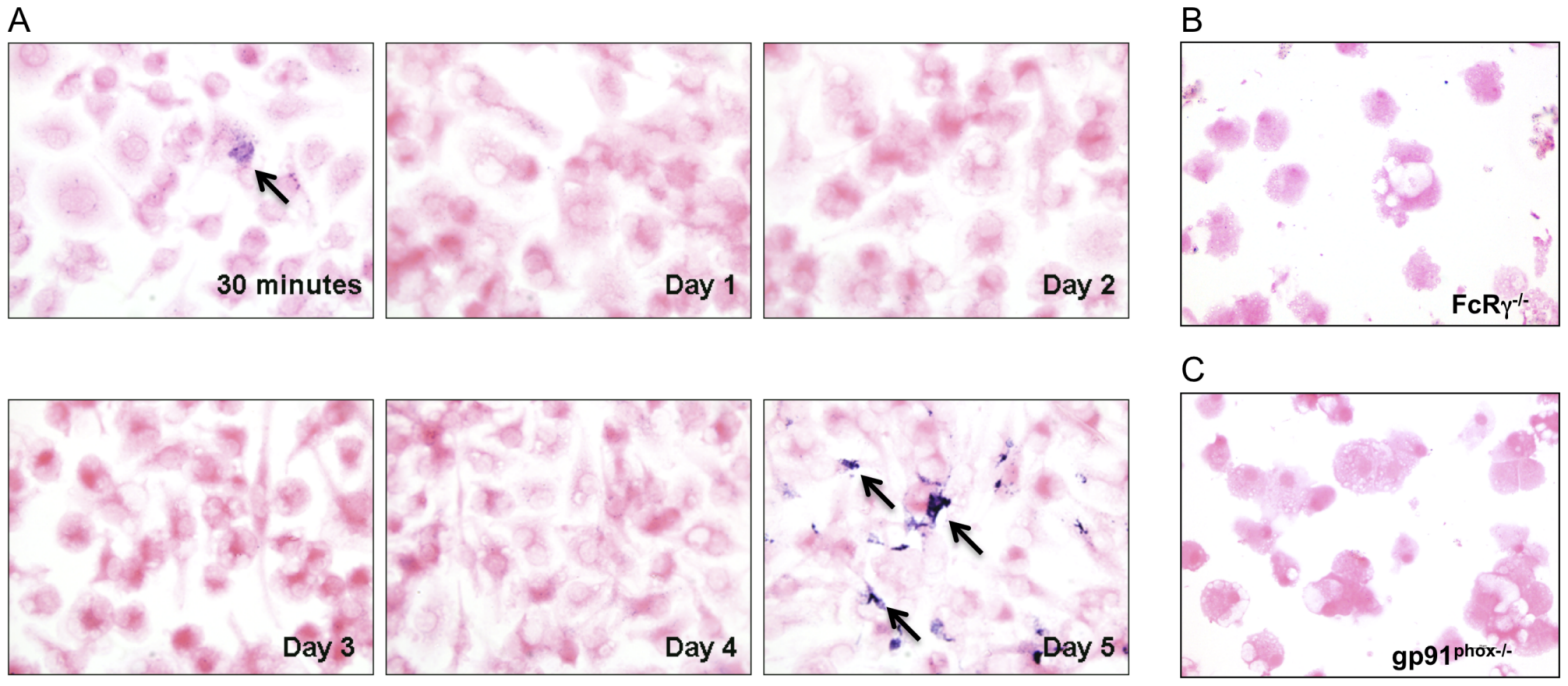
Superoxide production from *L. amazonensis* -infected BMM at day 5 post-immune cell activation. A) BMM from wild-type C57BL/6 mice were infected with *L. amazonensis* amastigotes and co-cultured with TLN cells from *L. major* infected mice. Cultures were incubated with nitro blue tetrazolamide (NBT) for 90 minutes at the time points indicated. Cells were counterstained with eosin and viewed using light microscopy at 40x magnification. Basophilic cytoplasmic precipitate (formazan) is indicative of superoxide production within infected cells (arrows). Results are representative of three separate experiments. B) BMM from mice deficient in FcRγ-common chain were treated as in A. Cultures were incubated with NBT for 90 minutes at 5 days post-activation. C) BMM from mice deficient in gp91^phox^ were treated as in A and incubated with NBT at day 5 post-activation. Results in B and C are representative of two separate experiments.

**Figure 3:**
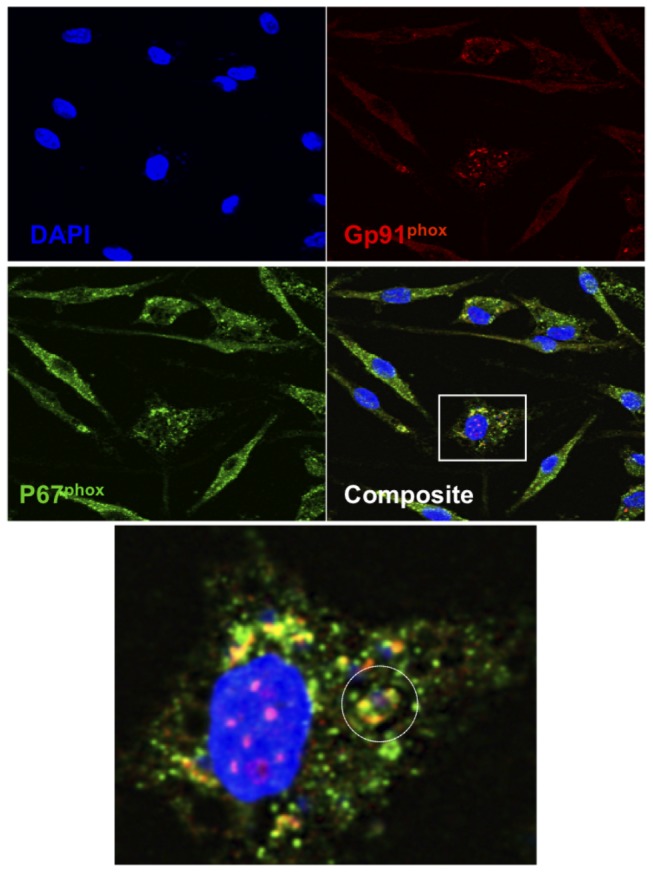
Immunofluorescent images of co-localized gp91^phox^ and p67^phox^. BMM were infected with *L. amazonensis* amastigotes and co-cultured with total lymph node cells from *L. major*-infected mice. Coverslips were recovered at day 5 post-activation, fixed and immunolabeled for gp91^phox^ and p67^phox^. A) Sequential scanning confocal microscopy (x60, oil) analysis of gp91^phox^ (red), p67^phox^ (green) and DNA (blue). Co-localization is represented in yellow (composite). White circle represents an amastigote nucleus (blue) surrounded by co-localized subunits (yellow). Data are representative of three separate experiments.

As shown in our previously published studies and in figure 1 above, reduction of parasite numbers occurred at day 5 of co-culture during in vitro cross-protection. To test the hypothesis that superoxide production coincided with parasite killing we assessed superoxide-dependent formazan precipitation by adding nitroblue tetrazolium (NBT) to the cultures at daily time points. While a small amount of formazan precipitate was observed early (30 minutes post-activation), readily detectable formazan only occurred at day 5 during the co-culture with 34 (+/−9.77 SEM) formazan precipitates per 100 cells, which corresponded to killing of *L. amazonensis* in infected BMM (Fig. 2A). To determine if this delayed superoxide production was dependent upon both FcγR signaling and NADPH oxidase function we used BMM from FcRγ-common chain deficient or gp91^phox^ deficient mice, respectively in this assay. Few, if any, (0–4) Formazan precipitates were detected in any of these genetically deficient BMM at any timepoint, including day 5 (Fig. 2 B and C). To our knowledge, this delayed intracellular production of superoxide in response to activation has not been previously defined. Therefore we wanted to confirm that the NADPH oxidase complex was assembled near the parasite at this late timepoint. Again utilizing our in vitro co-culture assay we harvested *L. amazonensis*-infected BMM following co-culture at day 5 and performed immunofluorescence analysis of NADPH subunits and parasite nuclei. In Figure 3 we show via confocal microscopy that at day 5 of co-culture, we find co-localization of gp91^phox^, a membrane bound subunit, and p67^phox^, a cytosolic subunit, with a Pearson's correlation coefficient of co-localization of 0.82. These co-localization events included areas near parasites based on nuclear DAPI staining. This subunit co-localization is consistent with the detection of superoxide at day 5.

### Superoxide production is dependent upon PI3K signaling

**Figure 4:**
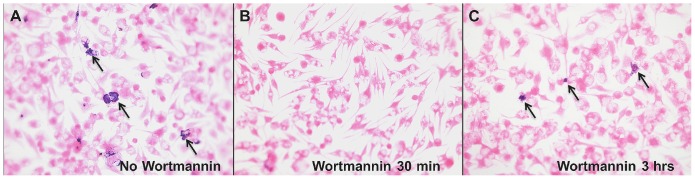
Wortmannin inhibits production of superoxide in BMM infected with *L. amazonensis*. BMM infected with *L. amazonensis* amastigotes and co-cultured with total lymph node cells from *L. major*-infected mice were incubated without (A) or with (B, C) 100nM wortmannin on day 5 post-lymphocyte activation. NBT was added after 30 mins (B) or 3 hrs (C) post-wortmannin treatment as indicated. Examples of formazan precipitate are indicated with arrows. Results are representative of two separate experiments.

FcγR-mediated activation of NADPH oxidase has been shown to be dependent upon PI3K signaling [22]. We hypothesized that generation of superoxide in *L. amazonensis*-infected BMM at day 5 reflected this pathway and was also PI3K dependent. To test this we added wortmannin, a PI3K inhibitor, to our in vitro system at day 5 post-activation and measured superoxide production via adding NBT to the cultures (Fig. 4A). If wortmannin was added 30 minutes prior to addition of NBT, no formazan precipitate was detected (Fig. 4B). We hypothesized that the effector molecules responsible for PI3K signaling persisted in the assay and that they were not limited to a relatively brief period of activation. The half-life of wortmannin is extremely short (10 minutes) [35] so to determine if the PI3K signaling for superoxide production was a continuous process we used the NBT assay 3 hours post-wortmannin treatment. As shown in Figure 4C formazan precipitates were again detected at this time (arrows).

### Soluble immune complexes (ICs) promote intracellular killing of *L. amazonensis*

**Figure 5:**
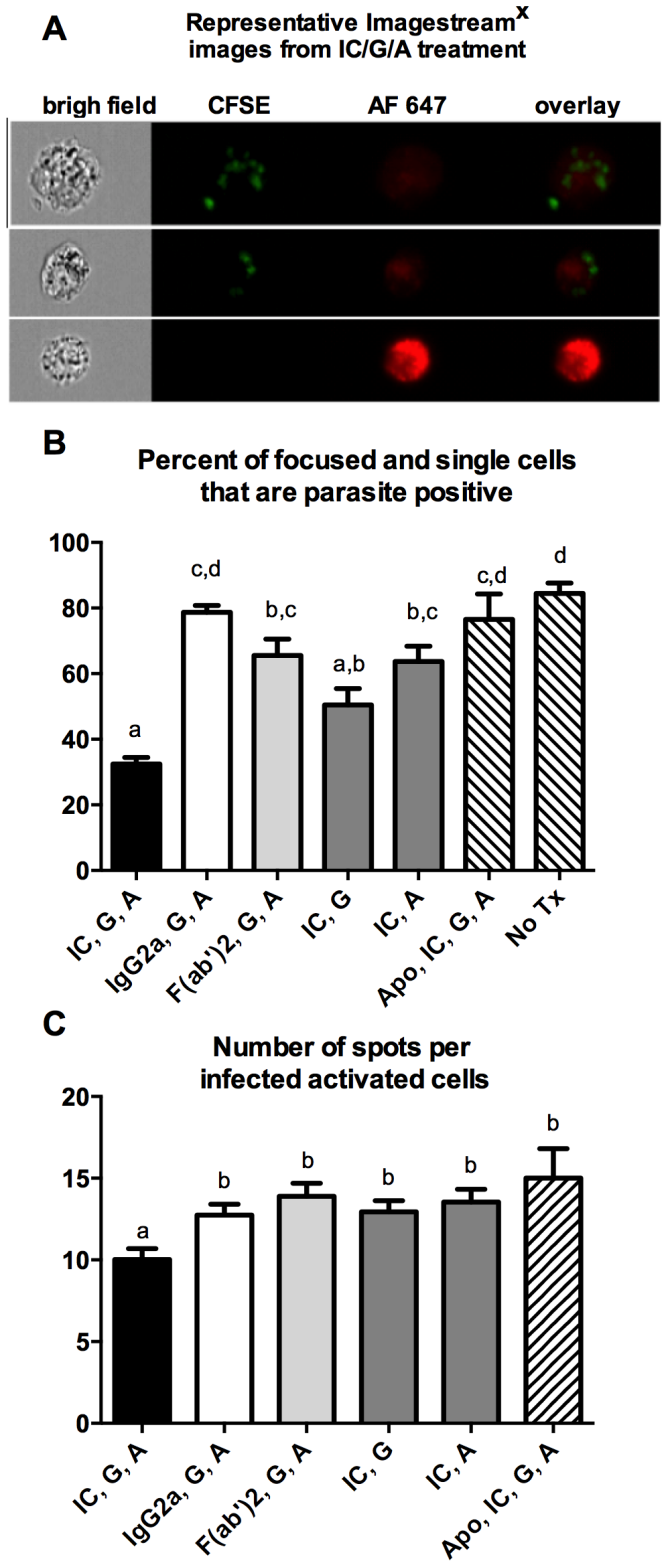
Soluble ICs kill *L. amazonensis* within infected macrophages. BMM were infected with CFSE-labeled *L. amazonensis* promastigotes. After 24 hrs the cells were washed and then activated as indicated in the graph. IC = soluble ICs; G = IFN-γ; A = Leishmania FT-Ag; IgG2a = monomer of IgG_2a_; Fab = F(ab)_2_ alone; Apo = apocynin; no Tx = no treatment. The cells were analyzed by ImageStream^X^ at 96 hours post-activation. A) Images are representative of a single analysis of IC, G, A, activated cells showing bright field of focused single cells, CFSE labeled parasites, and AlexaFluor647 (AF647) labeled soluble ICs and their overlay. B) Percentage of cells CFSE positive after indicated treatments. Different letters indicate significantly different means (p<0.05). C) Mean number of spots per CFSE positive cell under activating conditions. Different letters indicate significantly different means (p<0.05) from IgG2a, G, A, as the treatment control. B and C are the mean +/− SEM of pooled data from seven to two experiments. Statistical analysis was performed with Graph Pad's Tukey-Kramer multiple comparisons (B) or Dunnett's comparison to non-complexed IgG2a, IFN-γ, plus antigen as the control (C).

We previously demonstrated that in our in vitro cross-protection assay IgG2a antibodies are specifically required and we showed above that signaling through FcRγ–common chain was necessary for superoxide production and parasite killing (Fig. 1). We unsuccessfully tried to recapitulate antibody-FcγR activation pathways by adding immune serum from *L. major*-infected mice along with an IFN-γ source and parasite antigen to our in vitro killing assay (unpublished observations). We concluded that culture conditions specific to our assay must facilitate the generation of the antibody-dependent superoxide production. Given that antibodies would slowly accumulate in culture with antigen in excess, we hypothesized that soluble ICs might be the required component for delayed FcRγ-common chain-dependent macrophage activation. We therefore tested the ability of non-specific soluble IgG2a ICs to activate *L. amazonensis*-infected macrophages. To automate the in vitro killing assay and to determine if immune complexes directly associate with the intracellular parasite we used ImageStream^x^ analysis that uses a flow cytometry platform but additionally photo-records every event. Promastigote *L. amazonensis* parasites were stained with CFSE and non-specific murine IgG2a antibodies were labeled with AF 647. Figure 5A shows three cells from the activation conditions of soluble ICs, IFN-γ, and parasite antigen representing the fluorescent populations, CFSE bright (green-parasite), AF 647 bright (red-IgG2a), and dual positive cells. The ImageStream^x^ analysis showed in vitro killing by day 4 post-activation using three effector molecules; i) soluble ICs, ii) IFN-γ and iii) parasite antigen (Fig. 5B, first black bar). There was also a 20% reduction in the number of spots per cell compared to the control treatment group with non-complexed IgG2a, IFN-γ and antigen (p<0.05, Fig. 5C). These results confirmed similar experiments with all activation conditions quantitated microscopically (see Figure S1). Blocking NADPH assembly with apocynin prior to activation inhibited parasite killing (Fig. 5B, Apo, IC, G, A) and led to a 1.5 fold increase in the number of spots per cell (Fig. 5C, first and sixth bar). In the experiment represented in Figure 5A, out of 1672 single cell events analyzed, there were none that had a co-localized signal. However, co-localization signals varied between experiments and a correlation analysis revealed a weak positive relationship (R^2^ = 0.18, p<0.05) between co-localization of CFSE and AF 647 signals and the percentage of parasite positive cells in a treatment (data not shown).

## Discussion

Here we show that when *L. major*-induced cross-protection against *L. amazonensis* infection is recapitulated in vitro, parasite killing is dependent, in part, upon FcRγ-common chain-mediated NADPH oxidase activation. We find assembled NADPH oxidase complexes and PI3K dependent superoxide production at day 5 of the assay. This strongly suggests that the effector molecules required for superoxide production are generated during the co-culture conditions. Since parasite opsonization by antibodies is unlikely in this system we hypothesized that non-specific soluble ICs could recapitulate this antibody-FcRγ-common chain dependent NADPH oxidase activation. In testing this hypothesis we show that non-specific IgG2a soluble ICs, with IFN-γ and parasite antigen activate infected macrophages to kill *L. amazonensis* in a NADPH oxidase dependent process. Together with our previous studies these results support a model of *L. amazonensis* intracellular killing that is dependent, in part, on antibody-mediated activation of NADPH oxidase post-infection. Furthermore we can promote this mechanism of parasite killing using non-specific immune complexes in the co-culture system.

In experimental murine leishmaniasis antibodies are known to be associated with a non-productive immune response. Antibodies promote increased lesion size and parasite numbers during ineffective immunity towards *L. major*, *L. mexicana* and *L. amazonensis* [36], [37], [38]. High antibody titers after natural infection in humans and dogs are frequently associated with an uncontrolled infection [39]. Immunopathology, such as glomerulonephritis, is often associated with high levels of antibodies [40], [41], [42], [43]. There are only a few reports that indicate antibodies can have a positive outcome during *Leishmania* infection. Woelbing *et al* (2006) demonstrated that antibodies produced during productive immunity against *L. major* increased the efficiency of the immune response [44]. Recent studies have implicated *Leishmania*-specific B cells in enhancing T cell-mediated immunity during human disease [45]. Our laboratory has published that B cells and their IgG2a antibodies are required for killing *L. amazonensis* in our in vitro cross-protection assay [31]. We have also shown that after *L. amazonensis* infection C3H mice have a poor IgG2a response, whereas the same mice have a robust IgG2a response after *L. major* infection [32], [46]. One simple interpretation is that the B cell response reflects the ability of the immune system to either promote or exacerbate *Leishmania* infection. During ineffective immunity against *Leishmania* the B cell response promotes disease by limiting classical macrophage activation and promoting IL-10-mediated immunoregulation, whereas we propose that during effective immunity the B cell response associated with Th1 immunity can support healing, in part, by promoting NADPH oxidase-mediated superoxide production. During *L. major* infection this pathway is not required and the B cell response is dispensable. In contrast, *L. amazonensis* is resistant to the effects of nitric oxide alone and in our assay, B cells, and more specifically their production of antibodies, are required to promote superoxide production.

In our system, we demonstrate a novel mechanism whereby non-specific antibodies can trigger pathogen killing. This mechanism does require additional factors for activation. The requirement for IFN- γ is consistent with CD4^+^ Th1 cell-mediated immunity and IFN- γ induced nitric oxide production [21]. The requirement for killed *Leishmania* antigen is unexpected. We hypothesize that cellular pattern recognition receptors (PRRs) synergize with FcγRs to either influence signaling pathways and/or endosome trafficking patterns to target established parasitophorous vacuoles for NADPH oxidase assembly and activation. There are many examples of PRR synergism that can influence the macrophage response and this is a growing area of research [47], [48].

Gallo et. al. (2010) previously characterized how antibody concentration during opsonization can influence the macrophage response, with higher concentrations of antibodies promoting an immunoregulatory response that produces increasing levels of IL-10 [49]. Less is known of the immunomodulatory properties of ICs, although their involvement in inflammation is clear from their ability to promote the Arthus reaction and autoimmunity [50], [51]. A role for ICs in promoting a pro-inflammatory response during infection with intracellular pathogens is relatively unexplored. Studies by Pfefferkorn et. al. (1989) demonstrated that soluble ICs can lead to sustained superoxide production [27], [52]. Soluble ICs isolated from *Leishmania donovani*-infected patients have been shown to modulate macrophage responses in vitro with a significant increase in GM-CSF production [53]. Not surprisingly, the context of the soluble IC/macrophage interaction is important, as some studies using ICs have demonstrated IL-10-dependent immunoregulation [54], [55]. As recently discussed by Casadevall and Pirofski (2012) the plasticity of the antibody response makes it difficult to definitively demonstrate a positive role for antibodies during intracellular infections [56]. Again, we would suggest that during effective immunity against intracellular pathogens the B cell response is not just a bystander component, but actively supports the ability of the host to maintain low pathogen loads. The absolute requirement for this B cell response will vary both with the pathogen and the host.

Infection of C3HeB/FeJ mice with *L. major* can provide protection against concurrent or subsequent *L. amazonensis* challenge. Our studies utilize this cross-protection mechanism to determine what immune factors from *L. major*-infected mice can promote killing of *L. amazonensis* within infected macrophages. Here we clearly show the need for FcRγ common-chain and NADPH oxidase in killing *L. amazonensis*, in contrast to *L. major*. These results highlight the fact that different *Leishmania* species require different host antimicrobial mechanisms for effective control. We also show that non-specific soluble IgG2a ICs can have an indirect effect on intracellular parasite survival. This is similar to the finding that non-specific IgE ICs can kill *Leishmania* and *Toxoplasma gondii* via activation of the Fc**ε**II-CD23 receptor pathway in infected macrophages [57], [58]. These findings have implications for the immune dynamics of co-infections, as well as possible treatment strategies post-infection. In particular, these results suggest that therapies targeting the macrophage response against intracellular pathogens could be pursued through FcR pathways without having to identify pathogen specific epitopes. This pathway may be particularly relevant to situations where the B cell response is unable to generate effective antibodies [46]. Together the data from our in vitro analysis of cross-protection has uncovered a mechanism of macrophage activation effective against the intracellular parasite *L. amazonensis* that is partially dependent upon antibodies and which is functional post-infection.

## Supporting Information

Figure S1 **Soluble ICs kill** ***L. amazonensis*** **within infected macrophages (manual counts).** BMM were plated on glass coverslips and infected with *L. amazonensis* promastigotes. After 24 hrs the cells were washed and then activated as indicated in the graph. IC = soluble ICs; G = IFN-γ; A = Leishmania FT-Ag; IgG2a = monomer of IgG_2a_; Fab = F(ab)_2_ alone; Apo = apocynin; no Tx = no treatment. At 72 hours post-activation the cells were fixed and stained with HEMA 3 and examined by microscopy as described in Materials and Methods. A) The percentage of BMM containing parasites and B) the parasites per infected macrophage as a percent of IgG/G/A control. Results are the mean +/− SEM of 4 independent experiments. Different letters indicate significantly different means (p<0.05, Tukey-Kramer multiple comparisons).
(TIFF)

Data S1 **Data for** **Figures 1** **,** **2** **,** **5** **and S1.**
(XLSX)
