# Supplementary figures and images for: An In Vitro Model of Antibody-Enhanced Killing of the Intracellular Parasite Leishmania amazonensis
Source: PLoS One. 2014 Sep 5;9(9):e106426. doi: 10.1371/journal.pone.0106426 (PMC4156363; doi:10.1371/journal.pone.0106426)

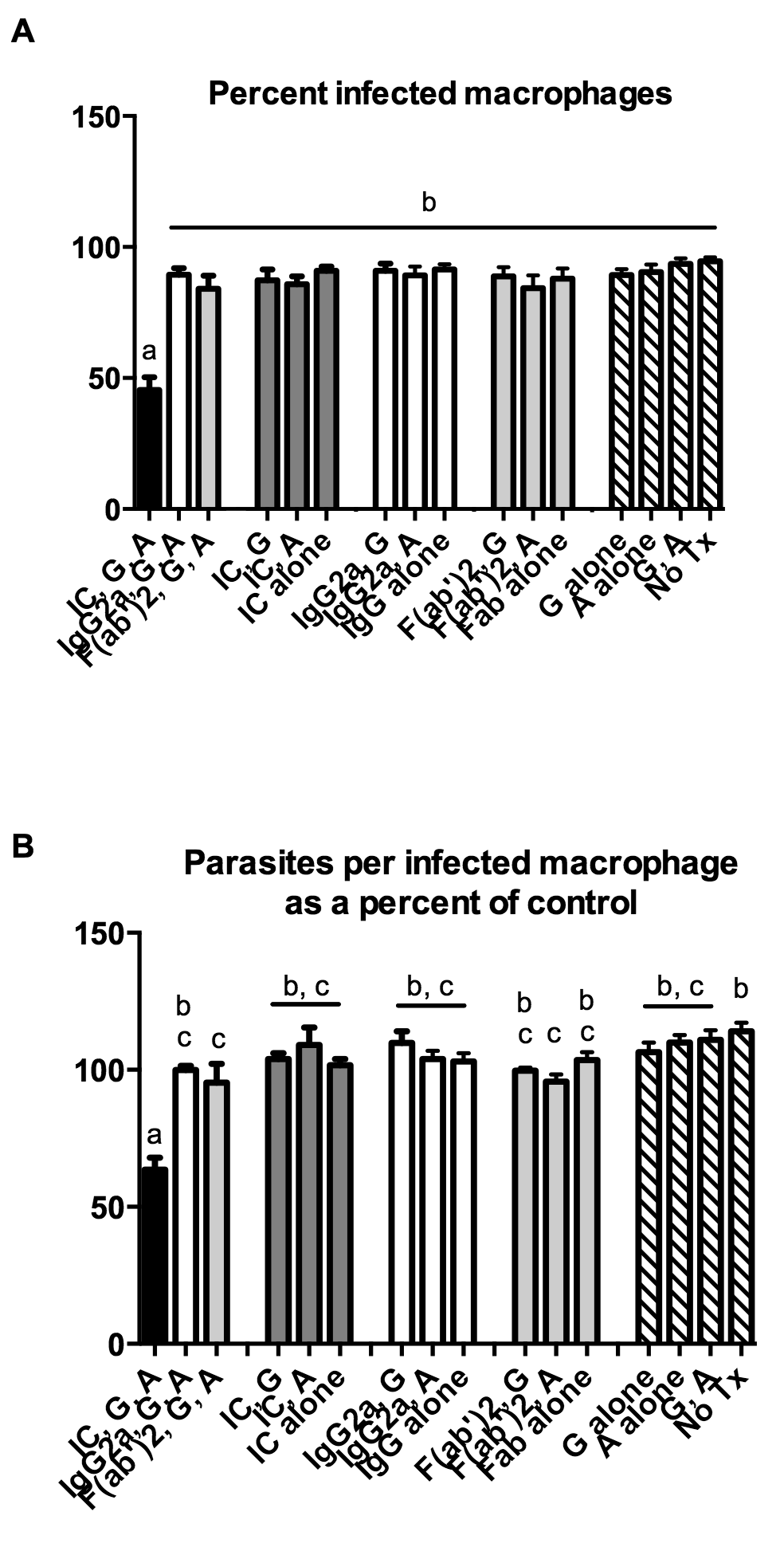

Supplement: Figure S1 — Soluble ICs kill L. amazonensis within infected macrophages (manual counts). BMM were plated on glass coverslips and infected with L. amazonensis promastigotes. After 24 hrs the cells were washed and then activated as indicated in the graph. IC = soluble ICs; G = IFN-γ; A = Leishmania FT-Ag; IgG2a = monomer of IgG2a; Fab = F(ab)2 alone; Apo = apocynin; no Tx = no treatment. At 72 hours post-activation the cells were fixed and stained with HEMA 3 and examined by microscopy as described in Materials and Methods. A) The percentage of BMM containing parasites and B) the parasites per infected macrophage as a percent of IgG/G/A control. Results are the mean +/− SEM of 4 independent experiments. Different letters indicate significantly different means (p<0.05, Tukey-Kramer multiple comparisons). (TIFF) [file pone.0106426.s001.tiff]
